# Does environmental regulation dividends inequality impact inclusive growth? Evidence from China

**DOI:** 10.3389/fpubh.2022.1061726

**Published:** 2022-11-16

**Authors:** Tao Ge, Ziqi Ding, Shuowan Lin, Yumeng Yang, Jianhua Ji

**Affiliations:** ^1^School of Economics and Management, Nantong University, Nantong, China; ^2^Jiangsu Yangtze River Economic Belt Research Institute, Nantong, China; ^3^China-ASEAN Institute of Statistics, Guangxi University of Finance and Economics, Nanning, China

**Keywords:** inclusive growth, threshold effect, transmission mechanisms, environmental regulation, dividends inequality

## Abstract

Based on the panel data of 281 city level in China for the period of 2004–2016, this study uses the Cobb–Douglas production function to investigate the distribution of environmental regulation dividends and further adopts the threshold model to explore the impact of environmental regulation dividends inequality (ERDI) on inclusive growth (IG). Results indicate that the distribution structure of the environmental regulation dividends has improved, but the inequality between urban–rural residents is still apparent. Environmental regulation dividends inequality has a non-linear threshold effect on inclusive growth, which turns from a significant inhibition to a slight promotion after exceeding the threshold value. Grouping tests show that environmental regulation dividends inequality has a heterogeneous effect on cities with different resource endowments and leading industries and still inhibits inclusive growth of non-resource-based cities even if the inequality is higher than the threshold value. Mechanism analysis reveals that primary distribution and redistribution are the main channels through which environmental regulation dividends inequality inhibits and promotes inclusive growth when the inequality is below and above the threshold value, respectively. These conclusions have important implications for enhancing and distributing environmental regulation dividends to promote inclusive growth.

## Introduction

China started economic reforms in the late 1970s and has experienced dramatic changes in the economy and society since then, which has lifted millions of Chinese people, especially rural residents, out of extreme poverty. However, China has been accompanied by long-term increase in the gap between the rich and the poor during this transition from a highly egalitarian collectivist model to a more open market economy. According to figures released by the National Bureau of Statistics, the income gap between the top 10% and the bottom 10% has increased from 7.3 times to 23 times during the period 1988–2007, and the per capita annual urban–rural household income gap ratio has increased from 2.57: 1 in 1978 to 3.23: 1 in 2010 (1). The Gini coefficient, which is widely employed to analyze income inequality, has also nearly doubled from 0.279 to 0.557 during the last decades (2). One of the priorities for alleviating income inequality is to follow the laws of social development to promote economic inclusiveness, which means that all people can share economic benefits more equitably (3). As such, promoting inclusive growth (IG) is an essential public concern and is of considerable significance to China.

China's extensive economic growth is not only accompanied by income inequality but also by serious environmental pollution (4). According to data released by the BP Statistical Review of World Energy, China not only surpassed the United States in 2006 to become the world's largest carbon dioxide emitter but also the largest energy growth driver, accounting for more than three-quarters of global net growth in 2019. Therefore, strengthening environmental governance has become an inevitable choice for the Chinese government. However, as an important part of social regulation, environmental regulation is not only a general tool for solving pollution problems but also an effective means for regulating income distribution (5). For example, with the introduction of the restrictive use policy of coal resources, the economic growth rate of Shanxi Province, China, with coal production as the main expenditure, has fallen sharply, and its ratio of per capita output and consumption to the national level has decreased from 85.1 and 77.4% in 2010 to 65.8 and 70.9% in 2016, respectively (6). These observations lead to the following questions: Can China's environmental regulation promote economic growth, that is, does China have environmental regulation dividends? Are China's environmental regulation dividends distributed equally among economic entities? If not, how does dividends inequality influence IG? What are the main channels of environmental regulation dividends inequality (ERDI) affecting IG?

The linkage between economic growth and income gap has been discussed for a long time, and the most important theoretical model is the Kuznets curve hypothesis. However, some scholars have found that the view of the Kuznets curve hypothesis is inconsistent with the determinants of income inequality, and then a more subtle view is put forward, that is, the government can choose different types of development patterns and different degrees of income inequality to achieve economic growth (7), which provides important empirical support for IG. IG is economic growth that pursues income equality among various segments (8), and its focuses are to increase development opportunities through rapid and sustainable growth and to ensure that the public can participate in and benefit from growth (9). The measurement of IG can be roughly summarized into three categories according to the research perspective. The first is to establish a social opportunity function from the perspective of social welfare and to select education and medical care as the equivalent of opportunity to measure economic inclusiveness (10). The second is to expand the concept of welfare function from the perspective of classical utilitarianism and construct a social mobility curve and Bonferroni curve to measure the inclusive index (11). The third is to introduce undesired output based on traditional total factor productivity from the perspective of efficiency to build inclusive total factor productivity (3, 12). Recent literature further explored the drivers of IG, which mainly focus on fiscal policy (13, 14), monetary policy (15, 16), structural policy (17, 18), and social policy (19, 20).

According to the double dividends hypothesis, environmental regulation has two potential positive effects reducing environmental pollution and increasing economic benefits (21), which has caused widespread discussion among environmentalists and policymakers (22). Cost–benefit analysis is a vital instrument to measure environmental regulation dividends because it can quantify costs and benefits in monetary terms (23). However, the costs and benefits of environmental policies may not occur at the same time, and the cost–benefit analysis used to assess the environmental regulation dividends is biased. The ex-post analysis avoids this shortcoming by dividing the sample into a treatment group and a control group based on whether or not to implement an environmental policy and comparing the two groups to identify the impact of the environmental policy (24). In addition, the computable general equilibrium model can simulate the effect of different policies or external shocks by modeling economic systems, and thus are widely used to assess environmental regulation dividends [e.g., (25, 26)]. The double dividends have been confirmed by existing literature [e.g., (27–29)], and according to different aspects of the economy, the second dividends have come in many forms, including employment dividends (30, 31), efficiency dividends (32, 33), health dividends (34, 35), and dividends distribution (36–38). However, some scholars hold a negative attitude toward dividends distribution of environmental regulation (39, 40). For example, Jiang and Shao (39) used Shanghai as an example to estimate the distribution effect of the carbon tax on households with different incomes and found that the carbon tax burden of low-income groups is the highest, which will aggravate income inequality.

With regard to the impact of ERDI on IG, recent literature has mostly focused on the linkage between environmental regulation and economic growth and offered three diametrically opposed views. Traditional neoclassical economics holds that environmental regulation will increase compliance costs and restrict the production efficiency and output growth of enterprises, which are not conducive to the sustainable development of a country or region [e.g., (41–44)]. At the end of the twentieth century, a group of scholars represented by Porter (45) introduced a dynamic innovation mechanism based on the static analysis framework and found that environmental regulation can stimulate enterprises to increase technological innovation and reduce production costs, and ultimately drive economic growth through technology diffusion and industrial upgrading [e.g., (46–48)]. Although the positive effect of environmental regulation on economic growth is supported by many empirical studies, some scholars question this view. For example, Kuosmanen et al. (49) pointed out that if a win–win situation between ecological protection and economic growth can be achieved through environmental governance, why should profit-seeking enterprises wait for environmental regulation to guide them? Therefore, some scholars argue that the impact of environmental regulation on economic growth may be uncertain [e.g., (50–52)].

The aforementioned literature not only extensively and in-depth investigates the connotation, measurements, manifestation, and determinants of IG and environmental regulation dividends but also systematically analyzes the linkage between environmental regulation and economic growth, which provides theoretical support and feasible methods for this study. However, although the aforementioned literature has confirmed the existence of environmental regulation dividends, it does not quantify the environmental regulation dividends. Meanwhile, all the discussion on environmental regulation dividends distribution also suggests that environmental regulation dividends may be unequal, which has not yet received widespread attention. In addition, although the link between environmental regulation and economic growth has been thoroughly investigated, the literature that incorporates environmental regulation and IG into a unified framework is rare.

This study, with a view to contributing to the previous work, will be expanded as follows: First, this study quantifies environmental regulation dividends and their distribution, and based on this, further investigates the impact of ERDI on IG, which not only contributes a novel perspective for exploring environmental economics but also improves the relevance of policy implications. Second, after the static linear analysis, this study further uses the dynamic panel threshold model to conduct an empirical test, which helps to accurately identify the features of ERDI affecting IG and provides an explanation for the debate on environmental regulation dividends distribution. Third, this study analyzes the impact and mechanisms of ERDI on IG, which provides a theoretical reference for similar research in the future. Fourth, economic activity mainly occurs in cities, and the use of city-level data can improve the reliability of estimates (53). Meanwhile, given the heterogeneity, this study divides cities according to resource endowment and leading industry and replaces core explanatory variables for the robustness test, which provides an empirical basis for policymakers to enhance and distribute environmental regulation dividends.

The remainder of this study is organized as follows: Section Mechanisms and hypotheses analyzes mechanisms and hypotheses. Section Methodology and data introduces the models and data utilized in this study. Section Results presents the empirical results and discussion. Section Conclusions concludes.

## Mechanisms and hypotheses

According to stakeholder theory, the environmental regulation dividends will be distributed among economic entities, including enterprises, governments, and residents. Enterprises are the main targets of environmental regulation. According to the “Porter Hypothesis”, environmental governance may increase the production cost of enterprises and make enterprises face greater competitive pressure, thus forcing enterprises to expand research and development investment and produce a “compensation effect” on technological innovation (54, 55). Obviously, this will improve the operating efficiency and profitability of enterprises and generates environmental regulatory dividends. However, small- and medium-sized enterprises have factors that are not conducive to green innovation, such as a shortage of innovation elements and excessive opportunity costs (56). Environmental regulation may force enterprises to move from innovative projects with development prospects to projects that reduce pollution (57), inhibit the initial development of enterprises, and reduce market vitality (58), which will reduce the environmental regulation dividends and the distribution of enterprises. Governments are makers and implementers of environmental regulation. Governments can not only obtain benefits through environmental penalties and green taxes but also can get environmental regulation dividends by levying business income tax on enterprises that create additional income due to environmental regulation. Residents are the main beneficiaries of environmental regulation. Environmental regulation can reduce environmental pollution and public health costs by restricting the negative external behaviors of enterprises, thereby solving the “environment-health-poverty” trap (59). Meanwhile, environmental governance will spur production activities related to energy saving, emission reduction, and resource utilization improvement, thereby promoting investment growth and scale development of environmental protection industries (60), which create more jobs than unemployment caused by unstable production and operation of enterprises (61), thus benefiting the overall employment status of society. However, environmental regulation mainly restricts pollution-intensive industries located in urban areas, so the reduction of urban residents' health expenditure is much higher than that of rural residents. Meanwhile, urban residents have inherent advantages in terms of employment skills, social networking, and information search, and they have more opportunities to obtain employment, especially in high-paying occupations. Therefore, this study proposes the following hypothesis:

Hypothesis 1: Environmental regulation will create dividends distributed among economic entities, but there is severe inequality between urban–rural residents.

Due to the urban–rural dual economic structure and long-term urban-biased policies, urban–rural residents have shown a clear income gap (62). As a result, when the environmental dividends gap between urban and rural residents is not apparent, it may not be noticed by policymakers, and the inhibition of ERDI on IG will be magnified with the increase in inequality. When the environmental regulation dividends gap between urban and rural residents exceeds a certain level, the inequality of urban–rural medical and health expenditures, environmental infrastructure, and ecological protection investment will become very prominent. To maintain healthy economic and social development, policymakers will implement rural-biased policies and use taxation, social security, and transfer payments as the main means of adjustment mechanisms to improve the distribution structure of urban and rural residents' environmental regulation dividends, thereby promoting IG. However, urban residents have a stronger preference for the environment and a higher willingness to pay (63). They can use information asymmetry to pay rural residents a certain amount of money and move rural residents away from areas with suitable environmental quality, which leads to the health benefits of urban residents expanding and environmental expenditures of rural residents increasing and forms an upside-down mechanism for the poor to subsidize the rich, thereby reducing the effectiveness of policies. Therefore, this study proposes the following hypothesis:

Hypothesis 2: There is a non-linear threshold effect on IG caused by ERDI that depends on the nexus between the inequality and the threshold value.

Under the market economy system, income distribution includes two basic levels: primary distribution and redistribution. The primary distribution is closely connected to the production factors, and it is formed mainly through market mechanisms that emphasize efficiency. Governments can adjust primary distribution using taxation and related laws and regulations. Redistribution is when governments adjust the income gap between economic entities through taxation, transfer payments, and social security after primary distribution, which emphasizes fairness more. Regarding environmental regulation dividends, when the inequality is below the threshold value, urban areas have first-mover advantages in terms of infrastructure, institutional environment, and labor quality, and capital profitability drives public health investment and environmental protection investment to urban areas and non-agricultural industries, which lead to insufficient investment in rural areas and agriculture. Therefore, primary distribution significantly widens the environmental regulation dividends gap between urban–rural residents. When the inequality is higher than the threshold value, governments will increase efforts to improve environmental regulation dividends distribution. It is, however, difficult to quantify environmental regulation dividends, and the primary distribution that uses taxation to regulate environmental regulation dividends distribution may not achieve the expected effect, which reduces the original intention of compensating rural residents. Redistribution can take advantage of rural-biased policies including public health expenditures, environmental governance investments, and environmental protection investments, to improve the profitability of rural residents from environmental regulation, and then play a corrective role in the inequality of environmental regulation dividends. Therefore, this study proposes that:

Hypothesis 3: When the inequality is lower and higher than the threshold value, ERDI mainly plays a negative and positive role in IG through primary distribution and redistribution, respectively.

## Methodology and data

### Proposed model

Income distribution is an old topic in economics, and the most classic income distribution model is the Cobb–Douglas production function with constraints (64). Therefore, this paper uses the Cobb–Douglas production function to construct an environmental regulation dividends distribution model.


(1) $$\begin{array}{l} erd_{it}=go{v_{it}}^{\alpha }en{t_{it}}^{\beta }ur{b_{it}}^{\theta }ru{r_{it}}^{\delta },i=1,2,\cdots \;,N;\\ \;t=1,2,\cdots \;,T. \end{array}$$

where *erd* denotes environmental regulation dividends; *gov* denotes the general budget revenue of governments; *ent* represents the total profit of industrial enterprises above designated size; *urb* represents the total disposable income of urban residents; and *rur* represents the total net income of rural residents; the subscripts *i* and *t* represent city and time, respectively. *α*, *β*, *θ*, and *δ* are the share of environmental regulation dividends obtained by economic entities, and there is a constraint *α* + *β* + *θ* + *δ* = 1.

Given that environmental regulation dividends cannot be measured directly, the technical function between environmental regulation dividends and input is set as: *erd*_*it*_ = *A*_*it*_*eri*_*it*_, where *A*_*it*_ is an unobservable technical and institutional factor. By logarithmic transformation of Equation (1), the following linear panel regression model can be obtained:


(2) $$\begin{array}{l} \ln eri_{it}=\alpha \ln gov_{it}+\beta \ln ent_{it}+\theta \ln urb_{it}\\ \;+\delta \ln rur_{it}+\phi_{it} \end{array}$$

where *ϕ*_*it*_ = ln (1/*A*_*it*_) is an unobservable technical and institutional parameters. To reflect the individual differences and time changes of technical and institutional factors, *ϕ*_*it*_ can be decomposed into individual effect *μ*_*i*_, time effect *f*_*t*_, and random factors *ε*_*it*_, and then Equation (2) can be converted into Equation (3):


(3) $$\begin{array}{l} \ln eri_{it}=\alpha \ln gov_{it}+\beta \ln ent_{it}+\theta \ln urb_{it}\\ \;+\delta \ln rur_{it}+\mu_{i}+f_{t}+\mu_{it} \end{array}$$

where a common factor *f*_*t*_ reflects the influence of technological progress or institutional change on different individuals. However, Equation (3) assumes that a common factor *f*_*t*_ has the same effect on different individuals, which is not in line with economic theory and empirical intuition. Therefore, referring to the practice of Bai (65), this study introduces the interaction term between time and individual to reflect the differences in individuals affected by the common factor *f*_*t*_, and then obtains Equation (4):


(4) $$\begin{array}{l} \ln eri_{it}=\alpha \ln gov_{it}+\beta \ln ent_{it}+\theta \ln urb_{it}\\ \;+\delta \ln rur_{it}+\mu_{i}+\nu_{t}+\lambda_{i}f_{t}+\varepsilon_{it} \end{array}$$

where λ_*i*_ reflects the heterogeneity of the common factor *f*_*t*_ in different individuals. The constraint *α* + *β* + *θ* + *δ* = 1 is transformed into *β* = 1-*α*-*θ*-*δ* and introduced into Equation (4), then Equation (5) can be obtained:


(5) $$\begin{array}{l} y_{it}=\alpha x_{1it}+\theta x_{2it}+\delta x_{3it}+\mu_{i}+\nu_{t}+{\text{λ}}_{i}f_{t}+\varepsilon_{it} \end{array}$$

where *y*_*it*_ = ln *eri*_*it*_ − ln *ent*_*it*_, *x*_1*it*_ = ln *gov*_*it*_ − ln *ent*_*it*_, ln *urb*_*it*_ − ln *ent*_*it*_, and ln *rur*_*it*_ − ln *ent*_*it*_.

According to the estimated parameters of Equation (5), this study can get technical and institutional factors and environmental regulation dividends. Furthermore, according to the environmental regulation dividends received by various economic entities, this study can analyze ERDI between urban–rural residents.

Based on the environmental regulation dividends of urban–rural residents, this study sets ERDI as $DI=\ln\frac{puerb}{prerb}$, where *puerb* and *prerb* represent per capita environmental regulation dividends of urban–rural residents, respectively. Obviously, the larger the *DI*, the greater the disparity between urban–rural residents. On this basis, this study investigates the impact of ERDI on IG, and constructs the model as follows:


(6) $$\begin{array}{l} IG_{it}=\varphi_{0}+\varphi_{1}DI+\varphi_{2}X+\mu_{i}+\nu_{t}+\varepsilon_{it} \end{array}$$

where *IG* represents inclusive growth, and *DI* is the core independent variable. X indicates a series of control variables affecting IG, including trade openness, physical capital, industrial structure, and unemployment. *μ*_*i*_ and ν_*t*_ represent individual effect and time effect, respectively, and *ε*_*it*_ is a random disturbance term. This study focuses on the coefficient of *DI*. If the coefficient is significantly positive, it indicates that ERDI promotes IG. Conversely, if the coefficient is significantly negative, it means that ERDI inhibits IG.

Considering that the impact of ERDI on IG may be non-linear, this study further uses the dynamic panel threshold model to investigate the dynamic characteristics of ERDI affecting IG. Referring to the research of (66), the model is set as follows:


(7) $$\begin{array}{l} IG_{it}=\varphi_{0}+\varphi_{1}IG_{it-1}+\varphi_{2}DI_{it}+\varphi_{3}X_{it}\\ \;+M1\left\{DI>\gamma \right\}+\mu_{i}+\varepsilon_{it} \end{array}$$

where *DI* represents the threshold variable and *γ* represents the threshold value. 1{•} indicates the indicator variable, and the value is 1 if *DI* > *γ*. The dependent variable is continuous before and after the inequality exceeds the threshold value, then Equation (7) is a kink model with *M* = *DI*_*it*_ − *γ*. The dependent variable is discontinuous before and after the inequality exceeds the threshold value, then Equation (7) is a jump model with *M* = *δ*_0_+*δ*_1_*IG*_*it*−1_+*δ*_2_*DI*_*it*_+*δ*_3_*X*_*it*_. One of the advantages of this method is that we do not judge the type of model in advance (67).

Finally, following the analytical framework used by Baron and Kenny (68), this study employs the medicating effect model to analyze the impact mechanism of ERDI, and sets the models as follows:


(8) $$\begin{array}{l} M_{it}=\rho_{0}+\rho_{1}M_{it\text{-}1}+\rho_{2}DI_{it}+\rho_{3}X_{it}\\ \;+M1\left\{DI>\gamma \right\}+\mu_{i}+\varepsilon_{it} \end{array}$$

(9) $$\begin{array}{l} IG_{it}=\eta_{0}+\eta_{1}IG_{it-1}+\eta_{2}M_{it}+\eta_{3}DI_{it}+\eta_{4}X_{it}\\ \;+M1\left\{DI>\gamma \right\}+\mu_{i}+\varepsilon_{it} \end{array}$$

where *M* represents the mediating variable, and the other parameters are defined above. According to the research of (3), there is a partial mediating effect if the signs of *ρ*_2_ × *η*_2_ and *η*_3_ are the same, and there is a masking effect if the signs of *ρ*_2_ × *η*_2_ and *η*_3_ are different.

### Inclusive growth

The connotation of IG stems from the integration of two economic and social goals, economic growth and income equality (8), and these two goals should not be separated because the trade-off between equity and efficiency may not exist (15). Therefore, this study constructs an inclusive total factor productivity index to denote IG based on an efficiency perspective. Input indicators include labor represented by social employment and capital stock measured by the perpetual inventory method, output indicators include good output represented by gross regional product using 2004 as the base year, and bad output represented by the urban–rural Theil index. The specific process is described in Ge and Li (3) and Ge et al. (69).

### Environmental regulation input

Following the research of (70), this study selects five single indicators to calculate environmental regulation input using the entropy weight method: the industrial sulfur dioxide removal rate, the industrial smoke (dust) removal rate, the industrial solid waste comprehensive utilization rate, the centralized treatment rate of the sewage treatment plant, and the harmless treatment rate of domestic waste. The detailed steps are as follows: First, the five single indicators are standardized: $pt_{ij}^{s}=\left[pt_{ij}-\min\left(pt_{j}\right)\right]/\left[\max\left(pt_{j}\right)-\min\left(pt_{j}\right)\right]$, where *pt*_*ij*_ represents the original value of the indicator *j* of city *i*, max(*pt*_*j*_) and min(*pt*_*j*_) represent the maximum and minimum of indicator *j*, respectively. Second, the information entropy of every single indicator is calculated: $E_{j}=-K\sum_{i=1}^{m}p_{ij}\ln p_{ij}$, where *K* = 1/ln *m* is the adjustment coefficient, *m* is the sample capacity, and *p*_*ij*_ is the proportion of the original value of indicator *j* of the city *i* in the country. Finally, according to the information entropy *E*_*j*_ and the weight of indicators *w*_*ij*_ = 1 − *E*_*j*_/*n* − ∑ *E*_*j*_(*j* = 1, 2, 3, ⋯ , *n*), environmental regulation input $ER_{ij}=\sum_{j=1}^{n}w_{j}pt_{ij}^{s}$ is calculated.

### Other variables

Referring to (69), the following factors are introduced as control variables: Trade openness (*TO*) is expressed by the ratio of import and export volumes in the gross regional product; industrial structure (*IS*) is represented by the proportion of the added value of the non-agricultural industry in the gross regional product; unemployment rate (*UR*) is represented by the proportion of registered urban unemployment in the local population; physical capital (*PC*) is expressed as the proportion of fixed-asset investment in the gross regional product.

According to the mechanism analysis above, the ERDI may act on IG through primary distribution and redistribution. Considering that wages can reflect the contribution of labor factors, this study uses employee wages (*WE*) as a substitute variable for primary distribution. Per capita GDP can reflect the final income level of a region, and its growth relative to employee wages can be roughly regarded as income transfer caused by redistribution. Therefore, this study uses income transfer (*IT*) as a substitute variable for redistribution.

Restricted to data availability and consistency, the sample used in this study consists of a panel dataset for 281 cities in China covering the period from 2004 to 2016, which leads to a total of 3,653 observations. The data used are obtained from the CEIC China Economic Database, the annual province-level or city-level statistical yearbook, and the national economic and social development statistical bulletin. After 2016, the China City Statistical Yearbook no longer counts “industrial sulfur dioxide production” and “industrial smoke (dust) production”, which makes it impossible to calculate the industrial sulfur dioxide removal rate and industrial smoke (dust) removal rate, thus making the data up to 2016.

## Results

### Analysis of environmental regulation dividends inequality

**Figure 1:**
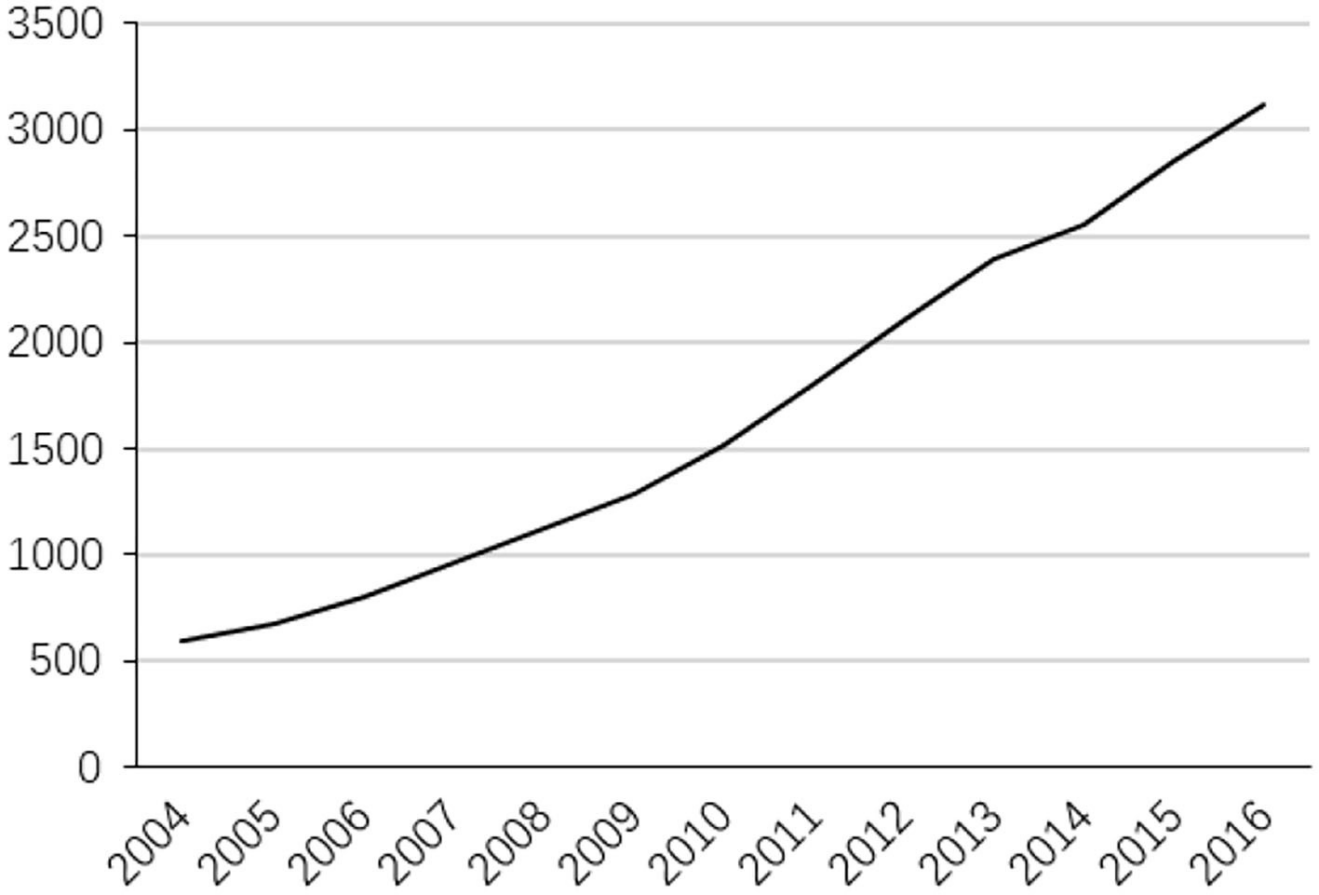
Environmental regulation dividends.

**Figure 2:**
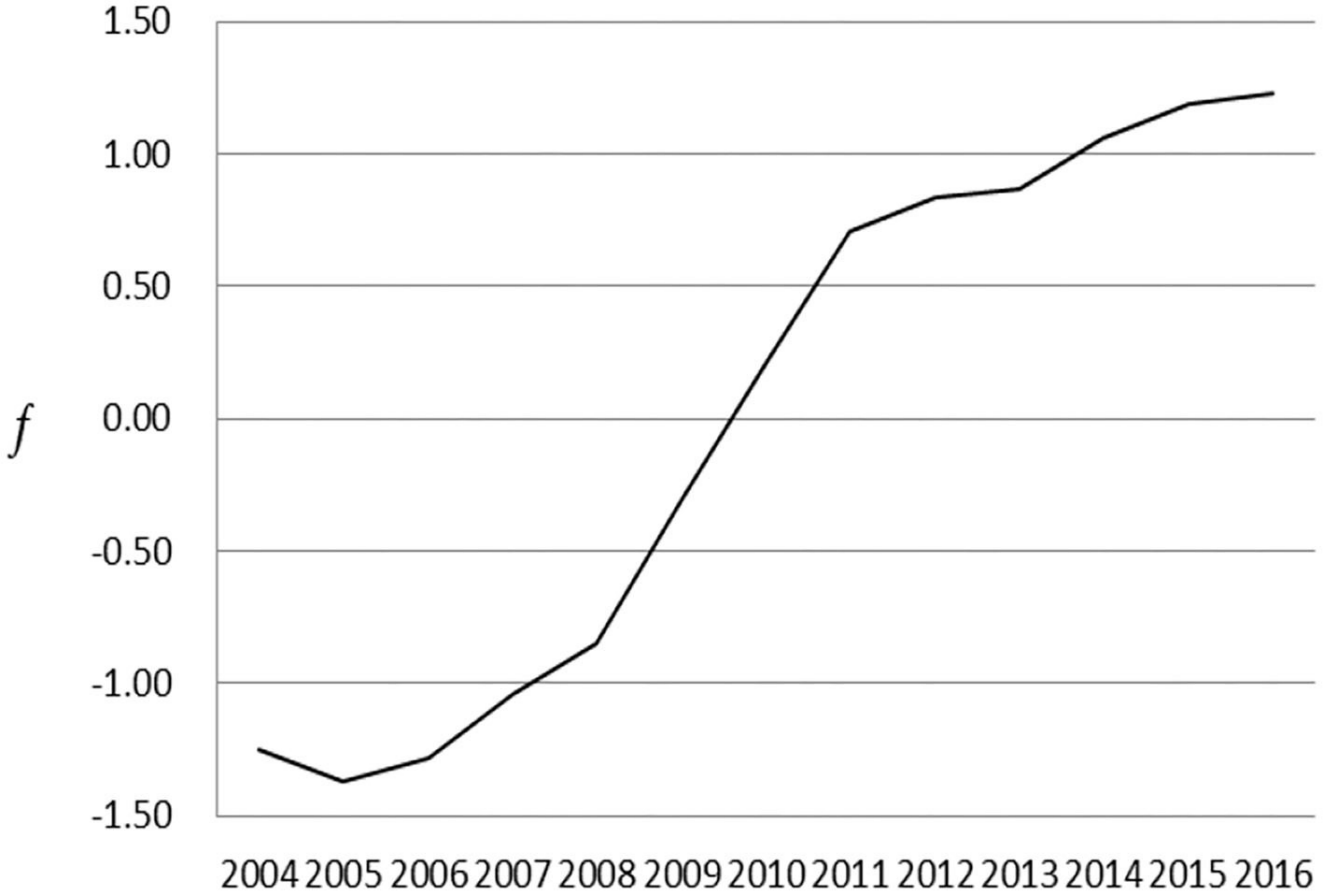
Common factor effect.

Figure 1 reports the annual environmental regulation dividend. It can be found that it has increased year by year, from less than 60 billion yuan in 2004 to more than 300 billion yuan in 2016. This may be related to the increasing intensity of China's environmental regulation. Figure 2 reports the results of technology and institution as a common factor. As a whole, the common factor showed a continuous upward trend, which illustrates the promotion effect of technological innovation and institutional changes on environmental regulation dividends. Specifically, the impact of the common factor on environmental regulation dividends from 2004 to 2011 has shown a significant upward trend, and after 2011, the upward trend has become relatively flat. Therefore, the impact of technological innovation and institutional changes on environmental regulation dividends has a breakpoint in 2011, which may lead to structural mutation. On the one hand, the 18th National Congress of the Communist Party of China in 2012 included ecological progress construction into the “Five-in-One” layout of socialism with Chinese characteristics and put forward the ambitious goal of building a beautiful China, which has helped make the objectives more explicit, the system more sound, and the content more detailed in environmental policy. On the other hand, the 18th National Congress of the Communist Party of China also pointed out that “to achieve the development benefits shared by the people, the reform of income distribution system must be deepened” and called for “improving the proportion of residents” income in national income distribution and increasing the proportion of labor remuneration in primary distribution”. Meanwhile, in the relationship between efficiency and fairness, it is clearly stated that “primary distribution and redistribution must take into account both efficiency and fairness, and redistribution pays more attention to fairness”. Fairness has been positioned more prominently than before, which reduces the incentive effect of technology and institutions on environmental regulation dividends.

Table 1 reports environmental regulation dividends distribution among economic entities, in which the distribution of governments and residents is obtained directly from the estimated coefficients, and the distribution of enterprises is obtained from the constraint between coefficients. Before 2011, the distribution ratios of environmental regulation dividends among governments, enterprises, and urban and rural residents were 26.29, 29.08, 43.69, and 0.94%, respectively. Obviously, urban residents get the most environmental regulation dividends, accounting for more than 40% of the total dividends, while the share of environmental regulation dividends obtained by rural residents is far lower than that of urban residents, which are consistent with our expectations. Most industrial enterprises are located in towns or suburbs (71), and urban residents have suffered more environmental pressures, so they enjoy relatively high environmental regulation dividends. The environmental regulation dividends of enterprises are second only to that of urban residents. Over the years, cheap natural resources have provided beneficial conditions for the development of enterprises but have also led to resource dependence. Environmental regulation has significantly improved the competitiveness and profitability of enterprises by promoting technological innovation, which has verified the “Porter hypothesis”. Governments' environmental regulation dividends are smaller than those of firms and residents. The essence of government is to serve residents and enterprises. After obtaining environmental regulation dividends, governments will convert their dividends into residents' dividends through ecological protection investment and environmental infrastructure construction.

**Table 1:** Environmental regulation dividends distribution.

**Period**	**Governments**	**Enterprises**	**Urban residents**	**Rural residents**
2004–2011	0.2629^***^ (0.0319)	0.2908	0.4369^***^ (0.0419)	0.0094^***^ (0.0069)
2012–2016	0.3336^***^ (0.0297)	0.0025	0.4025^***^ (0.0246)	0.2614^***^ (0.0232)
Changes	0.0707	−0.2883	−0.0344	0.2520

*** Represents significance levels of 1%; standard errors in parentheses. The same is below.

After 2011, the distribution of environmental regulation dividends changed significantly. The distribution ratios of governments, enterprises, and urban and rural residents are 33.36, 0.25, 40.25, and 26.14%, respectively. It can be found that environmental regulation dividends of urban residents have decreased by 3.44% points, and rural residents have increased by 25.2% points. This is mainly due to the policy orientation of the 18th National Congress of the Communist Party of China in 2012. The environmental regulation dividends of enterprises have decreased by 28.83% ge points, while those of governments have increased by 7.07% points. The 18th National Congress of the Communist Party of China put forward the requirements of “advancing green development, circular development, low-carbon development” and “building a beautiful China” for the first time, and China has entered the stage of comprehensively deepening environmental regulation reform. However, due to many factors such as enterprise scale, financial resources, and energy-saving and emission-reduction technology, small- and medium-sized enterprises can only choose to increase the passive expenditure such as emission fees, fines, and compensation, which causes a significant decline in enterprises' environmental regulation dividends and the increase in governments' environmental regulation dividends through environmental taxes. In summary, although the distribution structure of China's environmental regulation dividends among economic entities has improved since 2011, environmental regulation dividends of rural residents are still much lower than that of urban residents, which indicate that there is severe inequality in China's environmental regulation dividends between urban–rural residents and validates Hypothesis 1.

### Static effect analysis

Given the possibility of mutual causality between ERDI and IG, this study uses *DI*_*i, t*−1_ as the instrumental variable of *DI*_*i, t*_ to estimate the static effect, and the results are shown in Table 2. Model (1) does not include control variables. The results show that the Kleibergen–Paap rk LM statistic is 8.452, which rejects the null hypothesis that the instrumental variable has under-identification at a significance level of 1%. The Kleibergen–Paap rk Wald F statistic is 8,813.891, which is larger than the critical value of 16.38, indicating that there is a high correlation between the instrumental variable and endogenous variable, and rejecting the null hypothesis that the instrumental variable has weak identification. The Hansen J statistic is 0, and the null hypothesis cannot be rejected that the instrumental variable does not exhibit overidentification. The coefficient of *DI* is −0.0121 at the level of 1%, which indicates that ERDI is not conducive to IG. Models (2)–(5) add control variables, time, region, and province dummy variables based on Model (1). The results show that the instrumental variable still passes the validity test and that *DI* is significantly negative at least at the 10% level. Therefore, the inhibition of ERDI on IG is very robust.

**Table 2:** Static effect analysis.

**Variable**	**IG**				
	**(1)**	**(2)**	**(3)**	**(4)**	**(5)**
DI	−0.0121^***^ (0.0027)	−0.0135^***^ (0.0030)	−0.0074^**^ (0.0030)	−0.0072^**^ (0.0031)	−0.0078^*^ (0.0042)
_cons	0.9996^***^ (0.0114)	1.0432^***^ (0.0125)	0.9852^***^ (0.0146)	0.9895^***^ (0.0152)	1.0008^***^ (0.0231)
Controls	No	Yes	Yes	Yes	Yes
Time dummy	No	No	Yes	Yes	Yes
Region dummy	No	No	No	Yes	Yes
Province dummy	No	No	No	No	Yes
Kleibergen–Paap rk LM	8.452	7.969	8.001	7.723	4.966
Kleibergen-Paap rk Wald F	8,813.891	7,127.960	6,518.922	6,241.741	3,655.941
Hansen J	0.000	0.000	0.000	0.000	0.000
*N*	3,372	3,372	3,372	3,372	3,372
Adj-*R*^2^	0.0085	0.0299	0.1117	0.1154	0.1265

Standard error is in parentheses.

*** ,

** , and

* indicate significance levels at 1%, 5% and 10%, respectively.

### Dynamic effect analysis

After identifying the static effect, this study uses the dynamic panel threshold model to explore the dynamic effect of ERDI on IG. Table 3 provides the empirical results. Model (1) reports the result at the national level. The coefficient of *IG*_*t*−1_ is −0.1338 and passes the significance test of 1%, which shows that the habit effect restricts IG. The reason is that path dependence will result in pattern worship, and it is difficult to break the current GDP-oriented economic pattern and the policy limitation of IG. The threshold value is −4.3498, and passes the significance test of 1%, indicating that the model is a kink model. The coefficients of DI and kink are −0.5093 and 0.6185, respectively, and both pass the significance test of 1%, indicating that ERDI has a non-linear threshold effect on IG. When the inequality is below the threshold value, ERDI will inhibit IG, and the coefficient is as high as −0.5093. When the inequality is higher than the threshold value, ERDI can promote IG, but the coefficient is only 0.1092. Therefore, this study validates Hypothesis 2.

**Table 3:** Dynamic effect analysis.

**Variable**	**(1)**	**(2)**	**(3)**	**(4)**
	**National**	**Eastern**	**Central**	**Western**
*IG*_*t*−1_	−0.2213*** (0.0008)	−0.3331*** (0.0045)	−0.0743*** (0.0052)	−0.0760*** (0.0077)
DI	−0.5093*** (0.0119)	−0.4777*** (0.0301)	−0.3369*** (0.0258)	−0.9107*** (0.0327)
Controls	Yes	Yes	Yes	Yes
Kink	0.6185*** (0.0121)	0.6114*** (0.0309)	0.4783*** (0.0319)	0.9612*** (0.0356)
γ	−4.3498*** (0.0107)	−4.1274*** (0.0216)	−4.3690*** (0.0202)	−4.6549*** (0.0121)
*N*	3,653	1,274	1,300	1,079

*** indicates significant levels at 1%.

Models (2)–(4) report results at the regional level. It is not difficult to find that ERDI has a non-linear threshold effect on IG, but the impact on different regions varies. For example, when the inequality is lower and higher than the threshold value, ERDI has the most significant inhibition and the weakest promotion on IG of the western region, respectively. The proportion of employees engaged in resource-intensive industries in the western region is higher than that in the eastern and central regions, and environmental regulation dividends will cause a broader range of income fluctuation. Meanwhile, enterprises in the western region severely lack technological innovations, and their ability to create environmental dividends is also lacking. According to the threshold value at the regional level, this study analyzes changes in inequality. From 2004 to 2016, cities with inequality higher than the threshold value in the eastern, central, and western regions increased year by year, from 149, 225, and 189 in 2004 to 171, 251, and 228 in 2016, accounting for 13.42, 19.31, and 21.13% of the regional cities, respectively. Clearly, inequality is lower in most cities than the threshold value, which has a significant inhibition on IG. Meanwhile, given that inequality becomes increasingly severe, it is necessary for policymakers to breakdown path dependence and improve institutional mechanisms for inclusive development.

### Heterogeneous effect analysis

To further investigate the heterogeneous effect of ERDI affecting IG, this study divides cities into resource-based and non-resource-based cities, industry-oriented and non-industry-oriented cities according to their resource endowment and leading industry. Columns (1)–(2) of Table 4 report the results by resource endowment. The threshold value for both resource-based and non-resource-based cities is significantly positive at the 1% level, indicating that both are kink models. The coefficients of DI of the two types of cities are −0.0722 and −1.3125, respectively, and the coefficients of kink are 0.2264 and 1.2953, respectively, and they all pass the significance test of 1%. It shows that ERDI has a non-linear threshold effect. When the inequality is lower than the threshold value, ERDI has a stronger inhibition on non-resource-based cities than the resource-based city; when the inequality is higher than the threshold value, ERDI still shows an inhibition on non-resource-based cities, but it has a promotion on resource-based cities. Resource-based cities generally have resource dependence, and the “compensation effect” of technological innovation in environmental regulation is more significant, which will drive uneven growth in residents' income and stimulate governments to use primary distribution and redistribution to adjust the income gap between residents. Therefore, excessive inequality in environmental regulation dividends will promote IG.

**Table 4:** Heterogeneous effect analysis.

**Variable**	**(1)**	**(2)**	**(3)**	**(4)**
	**Resource-based**	**Non-resource-based**	**Industry-oriented**	**Non-industry-oriented**
*IG*_*t*−1_	−0.4053^***^ (0.0050)	−0.2863^***^ (0.0021)	−0.2569^***^ (0.0013)	−0.2277^***^ (0.0268)
DI	−0.0722^***^ (0.0161)	−1.3125^***^ (0.0411)	0.6545^***^ (0.0157)	−0.4443^***^ (0.1393)
Controls	Yes	Yes	Yes	Yes
Kink	0.2264^***^ (0.0166)	1.2953^***^ (0.0398)	−0.5964^***^ (0.0158)	0.9557^***^ (0.1617)
γ	−3.8994^***^ (0.0651)	−4.8630^***^ (0.0066)	−4.7903^***^ (0.0071)	−4.6098^***^ (0.0320)
*N*	1,495	2,158	3,133	520

*** indicates significant levels at 1%.

Columns (3)–(4) in Table 4 report the results of the leading industry. The threshold value for industry-oriented and non-industry-oriented cities is significantly positive at the level of 1%, indicating that both are kink models. The DI coefficients of the two types of cities are 0.6545 and −0.4443, and the kink coefficients are −0.5964 and 0.9557, respectively, and they all pass the 1% significance test, indicating that there are non-linear threshold effects. When the inequality is lower than the threshold value, ERDI shows positive promotion and negative inhibition on IG of industry-oriented and non-industry-oriented cities; when the inequality is higher than the threshold value, ERDI promotes IG of both types of cities, and the effect on non-industry-oriented cities is more prominent than that of industry-oriented cities. The income gap of industry-oriented cities is higher than that of non-industry-oriented cities because of the changes in labor productivity, labor quality, and employment structure, and the accumulation of advantageous resources in cities caused by industrialization. Therefore, even if the inequality is below the threshold value, industry-oriented cities will take measures to adjust the distribution of environmental regulation dividends. In contrast, only when the inequality is higher than the threshold value and the urban–rural gap is significantly expanded, non-industry-oriented cities will take the initiative to take action, and because they do not have habitual path dependence, regulatory policies will not be locked in inefficient operations status.

### Transmission mechanism analysis

Column (1) in Table 3 shows that there is a non-linear relationship between ERDI and IG, indicating that the mediating effect is valid, and analysis needs to be conducted. Table 5 reports the results of primary distribution and redistribution as mediating variables. The threshold value of Models (1)–(4) is significantly negative at the level of 1%, indicating that the models all have non-linear threshold effects. When primary distribution and redistribution variables are used to make regression analysis on ERDI, the coefficients of DI and kink are both significant at the level of 1%, indicating that both primary distribution and redistribution have an indirect effect. Specifically, when the inequality is lower than the threshold value, the models have a partial mediating effect, indicating that ERDI inhibits IG through primary distribution and redistribution, and the proportion of mediating effect caused by primary distribution and redistribution in the total effect is 3.09 and 0.17%, respectively. When the inequality is higher than the threshold value, the models have a masking effect, indicating that ERDI promotes IG through primary distribution and redistribution, and the indirect effects of primary distribution and redistribution are 0.55 and 0.17% of the total effects, respectively.

**Table 5:** Transmission mechanism analysis.

**Variable**	**(1)**	**(2)**	**(3)**	**(4)**
	**WE**	**IG**	**IT**	**IG**
*Y*_*t*−1_	0.9600^***^ (0.0004)	−0.2253^***^ (0.0009)	−0.0039^***^ (0.0000)	−0.1854^***^ (0.0015)
DI	0.1523^***^ (0.0034)	−0.2453^***^ (0.0051)	−2.1785^***^ (0.0075)	−0.3717^***^ (0.0082)
WE		−0.1037^***^ (0.0012)		
IT				0.0004^***^ (0.0000)
Controls	Yes	Yes	Yes	Yes
Kink	−0.1447^***^ (0.0036)	0.3885^***^ (0.0055)	0.9488^***^ (0.0233)	0.5319^***^ (0.0081)
γ	−4.3642^***^ (0.0157)	−3.5441^***^ (0.0104)	−3.6904^***^ (0.0064)	−4.5234^***^ (0.0066)
*N*	3,653	3,653	3,653	3,653

*** indicates significant levels at 1%.

There are differences in the two transmission mechanisms that ERDI affects IG. When the inequality is lower than the threshold value, the estimated coefficient of DI caused by primary distribution changes more, indicating that the effect of ERDI on IG is more significant through primary distribution. A market-led primary distribution is characterized by individual production factors and their prices in the market, and urban residents who enjoy natural advantages in capital, technology, management, and labor quality will inevitably receive more environmental regulation dividends and worsen the current primary distribution structure. When the inequality is higher than the threshold value, the estimated coefficient of DI caused by redistribution changes more, indicating that the effect of ERDI on IG is more obvious through redistribution. As ERDI gradually becomes excessive, the governments will use tax returns, social security, and transfer payments as the main means to make a second distribution of environmental regulation dividends to promote the coordinated development of urban–rural areas. This “reverse regulation” with an obvious rural bias makes the redistribution plays a more significant role in promoting IG, which validates Hypothesis 3.

### Robustness test

In this section, this study selects population density, employee salary, and per capita college students as the original indicators to quantify informal environmental regulation input, and then investigates informal environmental regulation dividends inequality and its impact. Table 6 provides the estimation results. For informal environmental regulation dividends distribution, before and after 2011, the ERDI of economic entities are urban residents, enterprises, governments and rural residents, and urban residents, governments, rural residents, and enterprises in order from high to low, which is consistent with formal environmental regulation. However, it should be noted that the informal environmental regulation dividends inequality of urban–rural residents is more severe than that of formal environmental regulation. On the one hand, informal environmental regulation characterized by voluntary public participation will increase residents' environmental protection costs and reduce the enthusiasm of rural residents with low-income levels. On the other hand, informal environmental regulation largely depends on public environmental protection awareness; urban residents with higher population quality are more willing to participate in environmental governance, so they also get more environmental regulation dividends. For the impact of environmental regulation dividends inequality, the threshold value passes the significance test of 1%, indicating that the model is a kink model. The coefficients of DI and kink are −0.2157 and 0.4352, respectively, and both pass the significance test of 1%, indicating that there is a non-linear threshold effect of informal environmental regulation dividends inequality on IG. The impact of informal environmental regulation dividends inequality on IG will turn from negative inhibition to positive promotion after the inequality exceeds the threshold value, which is in line with the findings of formal environmental regulation. Therefore, the results of this study are very robust.

**Table 6:** Robustness test.

**Variable**	**2004–2011**	**2012–2016**	**Variable**	**IG**
Governments	0.1757^***^ (0.0161)	0.3595^***^ (0.0233)	L.IG	−0.2896^***^ (0.0010)
Urban residents	0.5685^***^ (0.0213)	0.3836^***^ (0.0194)	DI	−0.2157^***^ (0.0036)
Rural residents	0.0040^***^ (0.0036)	0.2565^***^ (0.0186)	Controls	Yes
Enterprises	0.2518	0.0004	kink	0.4352^***^ (0.0050)
_cons	−8.7058^***^ (0.0300)	−8.5794^***^ (0.0212)	γ	−4.5705^***^ (0.0081)
*N*	3,653	3,653	*N*	3,653

*** indicates significant levels at 1%.

## Conclusions

Under the influence of rapid industrialization and continuous urbanization, China's economy has made a brilliant achievement, but it has also caused an excessive income gap and serious ecological damage, which results in inclusive growth (IG) and environmental governance becoming a hot public topic. Compared with the existing literature, this study analyzes the distribution of environmental regulation dividends among economic entities for the first time, explores the non-linear impact and heterogeneity of environmental regulation dividends inequality (ERDI) on IG, and investigates the transmission mechanisms of ERDI on IG from the perspective of primary distribution and redistribution, to help policymakers promote IG by enhancing and distributing environmental regulation dividends.

Panel data from 281 cities during 2004–2016 are used for empirical analysis. The main conclusions are as follows: the distribution structure of environmental regulation dividends has improved since 2011, but the inequality between urban–rural residents is still obvious. Second, the ERDI has a non-linear threshold effect. When the inequality is lower than the threshold value, the ERDI significantly inhibits IG. When the inequality is higher than the threshold value, the ERDI slightly promotes IG. Third, the impact of ERDI on IG is heterogeneous. According to resource endowment, when the inequality is lower than the threshold value, ERDI has a stronger inhibition on non-resource-based cities than resource-based cities; when the inequality is higher than the threshold value, ERDI still shows an inhibition on non-resource-based cities, but it has a promotion on resource-based cities. According to leading industry, when the inequality is lower than the threshold value, ERDI shows positive promotion and negative inhibition for industry-oriented and non-industry-oriented cities; when the inequality is higher than the threshold value, ERDI promotes IG of both types of cities, and the effect on non-resource-based cities is more prominent than that of resource-based cities. Fourth, primary distribution and redistribution are the main channels through that ERDI restricts and promotes IG when the inequality is below and above the threshold value, respectively. Fifth, the inequality of informal environmental regulation dividends is more severe between urban–rural residents and also has a non-linear threshold effect on IG.

The conclusions mentioned contribute to policy implications. First, policymakers should improve the institutional arrangements related to environmental regulation and optimize the allocation of governments' environmental regulation dividends. On the one hand, policymakers should fully utilize science and technology funding resources, stimulate technological innovation among enterprises, and improve the profitability of these enterprises and overall environmental regulation dividends. On the other hand, policymakers should increase the environmental infrastructure and capital investment of rural areas, promote the rural green environmental protection industries, and expand rural residents' access to environmental regulation dividends. Second, policymakers should actively play the incentives of primary distribution and redistribution. Policymakers should construct a set of quantitative indicators for environmental regulation dividends and strengthen the dynamic analysis of environmental regulation dividends and their distribution. Meanwhile, according to the city's geographical location, resource endowment, leading industry, and income inequality, policymakers should select a better adjustment mechanism or combination of two adjustment mechanisms from primary distribution and redistribution, to form a timely correction system for environmental regulation dividends inequality. Finally, policymakers need to enhance the ability to create informal environmental regulation dividends. Policymakers should innovate the environmental governance pattern, establish a daily mechanism for the collection and feedback of residents' opinions, and unblock the channels for public participation in environmental protection, to enhance the incentives of informal environmental regulation for enterprises' technological innovation. Meanwhile, policymakers should establish a cost compensation mechanism and financial incentive mechanism for public participation in environmental governance to improve residents' enthusiasm and profitability in informal environmental regulation.

## Data availability statement

The original contributions presented in the study are included in the article/supplementary material, further inquiries can be directed to the corresponding authors.

## Author contributions

TG: software, methodology, writing-original draft, and project administration. ZD: writing-original draft, data curation, and writing—review and editing. SL: data curation and writing—review and editing. YY: methodology and writing-original draft. JJ: conceptualization, investigation, and supervision. All authors contributed to the article and approved the submitted version.

## Funding

This study was supported by the National Social Science Foundation of China (No. 21CJL016) and supported by the Guangxi First-Class Discipline Statistics Construction Project Fund (No. GJKY2022(01)).

## Conflict of interest

The authors declare that the research was conducted in the absence of any commercial or financial relationships that could be construed as a potential conflict of interest.

## Publisher's note

All claims expressed in this article are solely those of the authors and do not necessarily represent those of their affiliated organizations, or those of the publisher, the editors and the reviewers. Any product that may be evaluated in this article, or claim that may be made by its manufacturer, is not guaranteed or endorsed by the publisher.
